# Analysis of risk factors for parenteral nutrition-associated cholestasis in preterm infants: a multicenter observational study

**DOI:** 10.1186/s12887-023-04068-0

**Published:** 2023-05-20

**Authors:** Ya-sen Wang, Wei Shen, Qing Yang, Rong Lin, Li-xia Tang, Rui-miao Bai, Dong Yang, Juan Zhang, Yi-jia Zhang, Wen-ting Yu, Shi-rong Song, Juan Kong, Si-yu Song, Jian Mao, Xiao-mei Tong, Zhan-kui Li, Fan Wu, Xin-zhu Lin

**Affiliations:** 1 Department of Neonatology, Women and Children’s Hospital, School of Medicine, Xiamen university, Xiamen, 361003 China; 2 Xiamen Key Laboratory of Perinatal-Neonatal Infection, Xiamen, China; 3 Xiamen Clinical Research Center for Perinatal Medicine, Xiamen, China; 4 Department of Neonatology, Northwest Women and Children’s Hospital, Xian, 710061 China; 5 Department of Pediatrics, Peking University Third Hospital, Beijing, 100191 China; 6 Department of Pediatrics, Shengjing Hospital of China Medical University, Shenyang, 110000 China; 7 Department of Neonatology, The Third Affiliated Hospital of Guangzhou Medical University, Guangzhou, 510150 Guangdong China

**Keywords:** Parenteral nutrition-associated cholestasis, Preterm infant, Nutrition, Multiple oil-fat emulsions, Risk factor

## Abstract

**Background:**

It is proposed that the development of parenteral nutrition-associated cholestasis (PNAC) was significantly associated with preterm birth, low birth weight, infection, etc.; however, the etiology and pathogenesis of PNAC are not fully understood. Most of the studies examining PNAC-associated risk factors were single-center studies with relatively small sample sizes.

**Objective:**

To analyze the risk factors associated with PNAC in preterm infants in China.

**Methods:**

This is a retrospective multicenter observational study. Clinical data on the effect of multiple oil-fat emulsions (soybean oil-medium chain triglycerides-olive oil-fish oil, SMOF) in preterm infants were collected from a prospective multicenter randomized controlled study. A secondary analysis was performed in which preterm infants were divided into the PNAC group and the non-PNAC group based on the PNAC status.

**Results:**

A total of 465 cases very preterm infants or very low birth weight infants were included in the study in which 81 cases were assigned to the PNAC group and 384 cases were assigned to the non-PNAC group. The PNAC group had a lower mean gestational age, lower mean birth weight, longer duration of invasive and non-invasive mechanical ventilation, a longer duration oxygen support, and longer hospital stay (*P* < 0.001 for all). The PNAC group had higher respiratory distress syndrome, hemodynamically significant patent ductus arteriosus, necrotizing enterocolitis (NEC) with stage II or higher, surgically treated NEC, late-onset sepsis, metabolic bone disease, and extrauterine growth retardation (EUGR) compared to the non-PNAC group (*P <* 0.05 for all). In contrast with the non-PNAC group, the PNAC group received a higher maximum dose of amino acids and fat emulsion, more medium/long-chain fatty emulsion, less SMOF, had a longer duration of parenteral nutrition, lower rates of breastfeeding, higher incidence of feeding intolerance (FI), more accumulated days to achieve total enteral nutrition, less accumulated days of total calories up to standard 110 kcal/kg/day and slower velocity of weight growth (*P <* 0.05 for all). Logistic regression analysis indicated that the maximum dose of amino acids (*OR*, 5.352; 95% *CI*, 2.355 to 12.161), EUGR (*OR*, 2.396; 95% *CI*, 1.255 to 4.572), FI (*OR*, 2.581; 95% *CI*, 1.395 to 4.775), surgically treated NEC (*OR*, 11.300; 95% *CI*, 2.127 ~ 60.035), and longer total hospital stay (*OR*, 1.030; 95% *CI*, 1.014 to 1.046) were independent risk factors for the development of PNAC. SMOF (*OR*, 0.358; 95% *CI*, 0.193 to 0.663) and breastfeeding (*OR*, 0.297; 95% *CI*, 0.157 to 0.559) were protective factors for PNAC.

**Conclusions:**

PNAC can be reduced by optimizing the management of enteral and parenteral nutrition and reducing gastrointestinal comorbidities in preterm infants.

## Introduction

The survival rates of very preterm infants (VPI) and very low birth weight infants (VLBWI) have improved significantly with the advances in perinatal medicine and neonatal intensive care unit (NICU), which include respiratory support techniques, nutritional management strategies and use of antenatal steroids [1]. A study from the Chinese Neonatal Network (CHNN) showed that the survival rate following active treatment of VPI in China was 95.4%, but the survival rate without any major complications was only 57.2%, which appeared significantly lower than in developed countries [2]. Active enteral and parenteral nutrition support is an important preventive and curative measure that can be implemented to improve the prognosis of VPI [3]. Due to the immaturity of the digestive system, VPI often rely fully or partially on parenteral nutrition (PN) in the early postnatal period to meet the nutrient and energy requirements for growth. However, prolonged PN has a potential negative impact on preterm infants, increasing the risk of serious complications, such as necrotizing enterocolitis (NEC), late-onset sepsis (LOS) and bronchopulmonary dysplasia (BPD) [4]. It may also cause atrophy of the gastrointestinal mucosa, impaired gallbladder contraction and parenteral nutrition-associated cholestasis (PNAC). Available evidence suggests that PNAC develops as a result of multiple factors. PNAC is one of the most common serious complications linked to preterm infants with a prevalence of 8.1–36.0% [5–7], which draws more attention from neonatologists. PNAC can be reversed with early intervention conferring a good prognosis. However, failure to initiate the treatment of PNAC in a timely manner will accelerates the disease progression and can lead to liver fibrosis, liver failure and even death.

In recent years, a lot of research has been conducted on identifying risk factors and prevention measures associated with PNAC. It is believed that the occurrence of PNAC is related to preterm birth, low birth weight, infection, prolonged fasting, NEC, extrauterine growth retardation (EUGR), high intake of amino acids, intravenous fat emulsions, etc. However, the etiology and pathogenesis of PNAC are not yet fully understood. Most studies focused on identifying risk factors for PNAC are single-center studies with relatively small sample sizes. Therefore, our study provided a secondary analysis of data from a prospective multicenter study on the effect of soybean oil-medium chain triglycerides-olive oil-fish oil (SMOF) on clinical outcomes in preterm infants. This work further investigated the risk factors associated with the development of PNAC and provided a basis for the prevention and treatment of PNAC.

## Methods

### Study population and design

The clinical data were obtained from a prospective multicenter study on the effect of SMOF in preterm infants, which was conducted in five tertiary level A hospitals with NICU of level IIIB or above in China between January, 2021 and December, 2021. The study was approved by the Ethics Committee of the Women and Children’s Hospital of Xiamen University (Grant No. KY-2020–0086–01) and it was registered in the China Clinical Trials Registry (Registration No. ChiCTR2100041910) (10/01/2021). Written informed consent was obtained from the parents or guardians of preterm infants included in the study.

#### Inclusion criteria included

(1) gestational age of preterm infants at birth less than 32 weeks or birth weight less than 1500 g, (2) starting time of PN < 48 h, (3) duration of PN > 2 weeks, (4) born in our hospital or transferred to our hospital within 24 h after birth.

#### Exclusion criteria included

(1) congenital intrauterine infection, (2) congenital malformation or inherited metabolic disease, (3) maternal-infant blood group incompatibility and hemolytic disease, (4) death or non-medical discharge within 2 weeks after birth.

#### Discharge criteria included

(1) cure of the original disease with stable vital signs, (2) oral feeding with amount of milk reached 150 mL/kg/d, (3) weight 2000 g or more, (4) postmenstrual age (PMA) ≥ 36 weeks.

**Primary objectives**: the risk factors for PNAC among preterm infants.

**Secondary objectives**: incidence of PNAC in preterm infants of different gestational ages and birth weights.

**Fig. 1:**
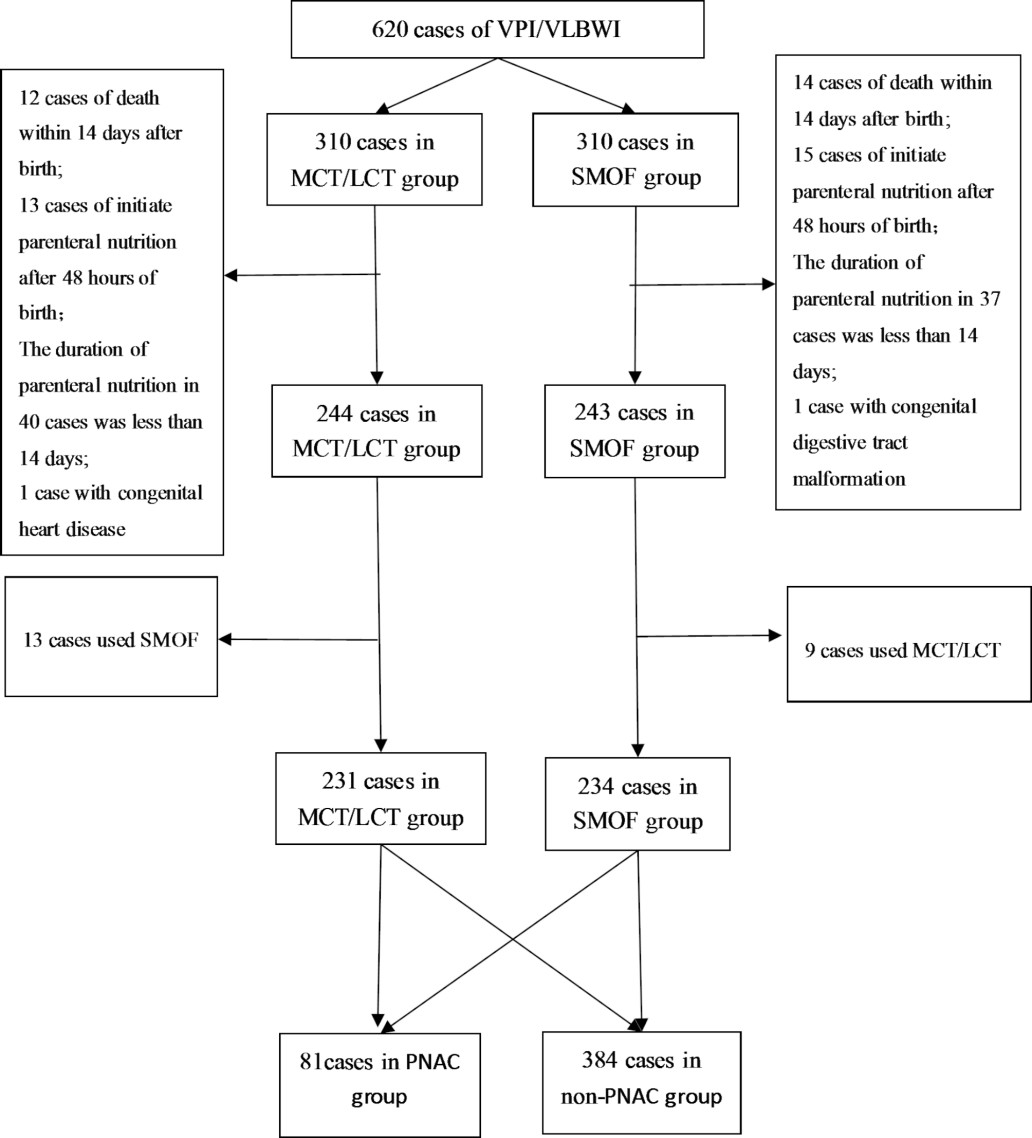
VPI, very preterm infant; VLBWI, very low birth weight infant; MCT, medium-chain triglycerides; LCT, long-chain triglycerides; PNAC, parenteral nutrition associated cholestasis

A total of 465 preterm infants were included in the study (Fig. 1). Based on the inclusion criteria, exclusion criteria and PNAC status, preterm infants were assigned to the PNAC group (n = 81) and the non-PNAC group (n = 384).

### Data collection

Collected data included general information on preterm infants and clinical data on early complications in preterm infants, including respiratory distress syndrome (RDS), hemodynamically significant patent ductus arteriosus (hsPDA), early-onset sepsis (EOS), stage II or higher NEC, surgically treated NEC, LOS, brain injury (including intraventricular hemorrhage and periventricular white matter softening), retinopathy of prematurity (ROP), moderate and severe BPD, metabolic bone disease of prematurity (MBDP ), EUGR, nutritional information related to the intake of amino acids and fat emulsions, duration of PN, time to initiate enteral feeding, breastfeeding, feeding intolerance (FI), time to reach total enteral nutrition and the number of days of total calorie intaked up to 110 kcal/kg/day.

### Study definition and diagnostic criteria

#### Diagnostic criteria

(1) PNAC is defined as PN lasting > 14 d with yellow skin staining and/or light stool color, direct bilirubin level ≥ 34 µmol/L (2 mg/dL) or direct bilirubin level ≥ 17umol/L (1 mg/dL) when total bilirubin level ≤ 85umol/L at any moment, and excluding any infections, such as viral, bacterial, and fungal, biliary tract developmental malformations and other causes [8]. (2) A PDA was classified as hemodynamically significant (hsPDA) when the ductus arteriosus was associated with ductal internal diameter > 1.5 mm, left atrial internal diameter/aortic internal diameter ≥ 1.4 or left ventricular end-diastolic internal diameter/aortic internal diameter ≥ 2.1, and with one of the following clinical manifestations, such as cardiac murmur, tachycardia (≥ 160 beats/minutes sustain), increased respiration, increased pulse pressure (> 25 mmHg), hypotension, water rushing pulse and cardiac enlargement [9]. (3) The severity of Bronchopulmonary dysplasia (BPD) was assessed at PMA 36 weeks according to the National Institutes of Health criteria, including (A) moderate, FiO_2_ 21-30%; (B) severe, FiO_2_ ≥ 30%; or requiring positive pressure ventilation or mechanical ventilation [10]. (4) EUGR was defined as weight below the 10th percentile of the 2013 Fenton curve for children of the same gestational age and sex at PMA 36 weeks or at discharge, [11]. (5) Clinical diagnostic criteria for EOS and LOS referred to the Expert Consensus on the Diagnosis and Treatment of Neonatal Sepsis (2019 edition) in China [12]. (6) Diagnostic criteria for MBDP were blood alkaline phosphatase > 900 IU/L with blood phosphorus < 1.8 mmol/L [13]. (7) ROP is classified according to the Guidelines for the Treatment of Premature Infants with Oxygen and Retinopathy (Revised) [14]; ROP requiring intervention means requires intravitreal drug injection, laser treatment or surgery. (8) Diagnostic criteria for RDS, NEC, brain injury, etc. referred to Practice of Neonatology (5th Edition) [15].

#### Relevant definition of intestinal nutrition and nutritional management

(1) Time of first-time milk feeding: initiation time of oral feeding of breast milk or formula after birth (excluding oral care using colostrum); (2) breastfeeding: the amount of breastfeeding for more than 50% of total enteral feeding; (3) time to achieve total enteral feeding: the time required to start enteral feeding until total enteral feeding reaches 150 mL/kg/d; (4) FI is defined as preterm infants inability to digest intestinal feeding and gastric retention exceeding 50% of the previous feed, development of abdominal distension and (or) vomiting, causing interrupted feeding schedules [16]; (5) nutritional management: all study subjects followed the 2013 Chinese clinical guidelines for neonatal nutrition support [17].

#### Calculation formula of weight growth velocity

The average velocity of weight growth (g/kg/d) = [1000 x ln(Wn/W1)]/(Dn-D1) [18].

Wn: the weight of the infant on the day of discharge (g),

W1: birth weight (g),

Dn: duration of hospital stay (days),

D1: the days required to regain birth weight (days).

#### Data management and quality control

The data entry clerks of each unit underwent uniform training before the start of the project and strictly followed the guidelines of the study protocol. EpiData software was applied to establish the database and the data of the case reports were entered after conducting consistency check by two doctors. Each participating unit collected data in real-time and uploaded relevant data after the infants were discharged from the hospital and the database was locked after verification and eliminating of errors. The technical staff of each unit kept close contact with the other participating unit at all times to check the case records, verify the accuracy of the data and solve any associated problems in a timely manner.

### Statistical methods

SPSS 22.0 software was used for statistical analysis. Enumeration data were expressed as rates (%) and comparisons between groups were made using the *χ*^*2*^ test or *Fisher’s* exact test. The *Kolmogorov-Smirnov* test was used to evaluate whether the measures conformed to a normal distribution. Normally distributed measures were expressed as *x ± s* and independent samples t-test was used for comparison between the groups. When the measurement data belong to abnormal distribution, data were presented as median (interquartile range) and the rank sum test was used for comparison between the groups. The correlations between birth weight, gestational age at birth, duration of PN and PNAC were calculated by Pearson’s correlation coefficient (*r*). Multivariate analysis was performed using binary logistic regression analysis. *p* < 0.05 was considered a statistically significant difference.

## Results

### Clinical information of premature infants between the two groups

Compared to the non-PNAC group, the PNAC group had a lower mean gestational age and weight at birth and longer duration of invasive and non-invasive mechanical ventilation, longer total oxygen support and longer total hospital stay (*p* < 0.001 for all). There were no significant differences in the incidence of low gestational age, sex, cesarean section, five-minute Apgar score ≤ 7, premature rupture of membranes > 18 h, etc. between the two groups (*P* > 0.05 for all) (Table 1).

**Table 1:** Comparison of clinical information of premature infants in the two groups

	Non-PNAC (n = 384)	PNAC (n = 81)	*Z/T/χ* ^*2*^	*P*
Mean gestational age at birth (weeks, $\bar {x} \pm s$)	30.1 ± 1.8	29.1 ± 2.2	4.007	<0.001
Mean birth weight (grams, $\bar {x} \pm s$)	1273.6 ± 248.2	1074.7 ± 279.7	6.406	<0.001
Male (n, %)	198(51.6)	47(58.0)	1.121	0.290
Cesarean section (n, %)	279(72.7)	57(70.4)	0.174	0.676
SGA (n, %)	53(13.8)	17(21.0)	2.701	0.100
Five-minute Apgar scores ≤ 7 (n, %)	37(9.6)	9(11.1)	0.163	0.686
Premature rupture of membranes > 18 h (n, %)	59(15.4)	11(13.6)	0.167	0.683
Invasive mechanical ventilation (days)(mean, IQR)	0.0(0.0,3.0)	3.0(0.0,10.0)	-4.263	<0.001
Non-invasive mechanical ventilation (days)(mean, IQR)	18.0(8.0,31.0)	33.0(15.0,49.8)	-4.220	<0.001
Total duration of oxygen support (days)(mean, IQR)	34.0(18.0,51.0)	52.5(34.8,68.0)	-5.186	<0.001
Maternal gestational diabetes (n, %)	99(25.8)	22(27.2)	0.066	0.797
Maternal hypertension in pregnancy (n, %)	117(30.5)	27(33.3)	0.257	0.612
Total hospital stay (days)(mean, IQR)	47.0(38.0,60.0)	67.0(53.0,82.0)	-6.573	<0.001

PNAC, parenteral nutrition-associated cholestasis; SGA, small for gestational age

### Occurrence of PNAC stratified by birth weight and gestational age

The incidence of PNAC in infants born at gestational age < 28w, 28–29w and > 30w were 34.2%, 16.7% and 12.7%, respectively. The incidence of PNAC in infants born at weight < 1000 g, 1000–1499 g and ≥ 1500 g were 42.3%, 13.8% and 5.8%, respectively. Lower gestational age and lower birth weight were associated with a higher incidence of PNAC (*r* values were − 0.180 and − 0.277, respectively) (Table 2).

**Table 2:** Comparison of the incidence of PNAC in different birth weight and gestational age groups

	Non-PNAC (n = 384)	PNAC (n = 81)	*χ* ^*2*^	*P*	Pearson’s correlation coefficient (*r*)
Gestational age at birth (n, %)	-				
< 28w	48(65.8)	25(34.2)	18.066	<0.001	-0.180^*1^
28 ~ 29w	130(83.3)	26(16.7)			
>30w	206(87.3)	30(12.7)			
Birth weight(n, %)	-				
< 1000 g	45(57.7)	33(42.3)	42.904	<0.001	-0.277^*2^
1000 g ~ 1499 g	274(86.2)	44(13.8)			
≥ 1500 g	65(94.2)	4(5.8)			

PNAC, parenteral nutrition-associated cholestasis. *1: *P =* 0.050; *2: *P* = 0.046

### In-hospital nutritional status between groups

Compared to the non-PNAC group, preterm infants in the PNAC group received more medium/long-chain fat emulsion and less SMOF. The incidence of PNAC was 13.7% in the preterm infant group receiving SMOF compared to 21.2% in the preterm infant group receiving medium/long-chain fat emulsion (*P =* 0.002). The duration of PN was longer for patients with a higher incidence of PNAC. The incidence of PNAC was 9.0%, 13.0% and 29.6% in preterm infants with duration of PN < 3w, 3w-4w and > 4w, respectively. The duration of PN was positively associated with the incidence of PNAC (*r* = 0.235, *P* = 0.044). In addition, the PNAC group had higher maximum doses of amino acids and fat emulsion, lower rates of breastfeeding, higher incidence of FI, more accumulated days to achieve total enteral nutrition, a slower velocity of weight growth and fewer accumulated days of total calories up to standard 110 kcal/kg/day (*P* < 0.05 for all). There were no significant differences in the initial dose of amino acid, fat emulsion, etc. between the groups (Table 3).

**Table 3:** Comparison of the in-hospital nutritional status of infants between the groups

	Non-PNAC (n = 384)	PNAC (n = 81)	*Z/T/χ* ^*2*^	*P*
Amino acid dose initiation (g/kg/day) (mean, IQR)	1.9(1.1,2.1)	1.5(1.,4, 1.9)	-1.935	0.053
Amino acids dose up to 3.0 g/kg/d (days)(mean, IQR)	4.0(3.0,5.0)	4.0(3.0,5.0)	-0.001	1.000
Maximum dose of amino acids (g/kg/day) (mean, IQR)	3.5(3.2,3.7)	4.0(3.5,4.0)	-7.331	<0.001
SMOF (n, %)	202(52.6)	32(39.5)	4.590	0.032
Medium/long-chain fat emulsion (n, %)	182(47.4)	49(60.5)	4.590	0.032
Fat emulsion dose initiation (g/kg/day) (mean, IQR)	1.0(1.0,1.5)	1.0(1.0,1.0)	-0.346	0.729
Fat emulsion dose up to 3.0 g/kg/d (days)(mean, IQR)	5.0(4.0,7.0)	5.0(4.0,7.0)	-1.648	0.099
Maximum dose of fat emulsion (g/kg/day) (mean, IQR)	3.0(3.0,3.0)	3.0(3.0,3.4)	-2.836	0.005
Days of parenteral nutrition (days)(mean, IQR)	25.0(18.0,32.0)	34.0(24.0,43.5)	-5.905	<0.001
Time to initiate enteral feeding (hours) (mean, IQR)	24.0(16.0,41.0)	24.0(18.0,42.8)	-1.793	0.073
Breast feeding (n, %)	209(54.4)	22(27.2)	19.893	<0.001
FI (n, %)	140(36.5)	56(69.1)	29.293	<0.001
Time to reach total enteral nutrition (days)(mean, IQR)	26.0(19.0,34.0)	35.0(24.0,44.8)	-4.169	<0.001
Total calories up to 110 kcal/kg/day (days)(mean, IQR)	12.0(8.0,18.0)	9.5(6.8,16.0)	-2.322	0.020
Days to regain birth weight (days) (mean, IQR)	10.0(8.0,12.0)	10.0(8.0,11.5)	-0.611	0.541
Average velocity of weight growth (g/kg/day) (mean, IQR)	15.2(13.6,17.2)	13.7(12.8,15.5)	-3.322	0.001

PNAC, parenteral nutrition-associated cholestasis; SMOF, soybean oil-medium chain triglycerides-olive oil-fish oil; FI, feeding intolerance

### Incidence of preterm complications in patients between the two groups

The incidence of RDS (P = 0.004), hsPDA (P *=* 0.015), stage II or more, surgically treated NEC (P = 0.004), LOS (P *=* 0.037), MBDP (*P* < 0.001) and EUGR (*P* < 0.001) was higher in the PNAC group compared to the non-PNAC group. There was no statistically significant difference in the incidence of EOS, brain injury, ROP with intervention and moderate to severe BPD between the groups (*P* > 0.05 for all) (Table 4).

**Table 4:** Comparison of preterm complications of patients between the two groups

	Non-PNAC (n = 384)	PNAC (n = 81)	*Z/T/χ* ^*2*^	*P*
RDS (n, %)	233(60.7)	63(77.8)	8.455	0.004
hsPDA (n, %)	209(54.4)	56(69.1)	5.904	0.015
EOS (n, %)	25(6.5)	9(11.1)	2.089	0.148
NEC ≥ stage II (n, %)	17(4.4)	11(13.6)	8.352	0.004*
Surgically treated NEC	5(1.3)	8(9.9)	15.079	<0.001*
LOS (n, %)	66(17.2)	22(27.2)	4.336	0.037
Brain injury (n, %)	137(35.7)	29(35.8)	<0.001	0.983
ROP with intervention (n, %)	13(3.4)	4(4.9)	0.123	0.726*
Moderate/severe BPD (n, %)	77(20.1)	16(19.8)	0.004	0.951
MBDP (n, %)	8(2.1)	10(12.3)	16.274	<0.001*
EUGR (n, %)	199(51.8)	62(76.5)	16.600	<0.001

*Continuity correction chi square test. PNAC, parenteral nutrition-associated cholestasis; RDS, respiratory distress syndrome; hsPDA, hemodynamically significant patent ductus arteriosus; EOS, early-onset sepsis; NEC, necrotizing enterocolitis; LOS, late-onset sepsis; ROP, retinopathy of prematurity; BPD, bronchopulmonary dysplasia; MBDP, metabolic bone disease of prematurity; EUGR, extrauterine growth retardation

### Multivariate logistic regression analysis affecting the occurrence of PNAC

All univariate results *at p* < 0.05 were included in logistic regression analysis, which showed that higher maximum doses of amino acids (*OR*, 5.352; 95% *CI*, 2.355 to 12.161), EUGR (*OR*, 2.396; 95% *CI*, 1.255 to 4.572), FI (*OR*, 2.581; 95% *CI*, 1.395 to 4.775), surgically treated NEC (*OR*, 11.300; 95% *CI*, 2.127 to 60.035) and longer total hospital stay (*OR*, 1.030; 95% *CI*, 1.014 to 1.046) were independent risk factors for the development of PNAC. SMOF (*OR*, 0.358; 95% *CI*, 0.193 to 0.663) and breastfeeding (*OR*, 0.297; 95% *CI*, 0.157 ~ 0.559) were protective factors for PNAC (Table 5).

**Table 5:** Multivariate logistic regression analysis affecting the occurrence of PNAC

	*Β*	*S.E.*	*P*	*OR* (95% *CI*)
SMOF	-1.027	0.315	0.001	0.358(0.193 ~ 0.663)
Maximum dose of amino acids	1.677	0.419	<0.001	5.352(2.355 ~ 12.161)
Breastfeeding	-1.215	0.324	<0.001	0.297(0.157 ~ 0.559)
EUGR	0.874	0.33	0.008	2.396(1.255 ~ 4.572)
FI	0.948	0.314	0.003	2.581(1.395 ~ 4.775)
Surgically treated NEC	2.425	0.852	0.004	11.300(2.127 ~ 60.035)
Total number of days in hospital	0.029	0.008	<0.001	1.030(1.014 ~ 1.046)

SMOF, soybean oil-medium chain triglycerides-olive oil-fish oil; FI, feeding intolerance; EUGR, extrauterine growth retardation; NEC, necrotizing enterocolitis

## Discussion

Infants with VPI or VLBWI are at higher risk of developing PNAC. This study showed that the incidence of PNAC was 17.4% in preterm infants, which is consistent with the study by Lee et al, 2016 [6]. PNAC not only causes hepatocellular damage, liver fibrosis, impaired absorption of fat-soluble vitamins, increased risk of bleeding disorders and bone loss but also has a detrimental effect on the long-term neurocognitive function of children [19]. Talcott et al. showed that prolonged cholestasis could impair neurological development [20]. They reported that children with PNAC had significantly lower Bayley-III scores for cognition, language and motor function at 12 and 24 months of corrected age compared to children without PNAC (*p* < 0.05), suggesting that PNAC is a predictor variables and potential risk factor for poor neurological prognosis in extremely low birth weight infants. Therefore, identifying the risk factors associated with PNAC is of great clinical significance to prevent or reduce the occurrence of PNAC and improve the prognosis of preterm infants in the near and long term.

### Effect of gestational age at birth and birth weight on PNAC

The present study showed that both gestational age at birth and birth weight were lower in the PNAC group (*p* < 0.001), and that the development of PNAC was negatively correlated with both gestational age at birth and birth weight (*r* values were *− 0*.180 and − 0.277, respectively). Lee et al. [6] showed that lower birth weight was an independent risk factor for PNAC. Zhang et al. [21] also reported that low gestational age at birth and low birth weight were the main risk factors for the progression of PNAC. However, in the present study, multivariate regression analysis showed that gestational age at birth and low birth weight were not independent risk factors for PNAC. A retrospective study of risk factors associated with PNAC in preterm infants showed that birth weight and gestational age at birth were not independent risk factors for PNAC in preterm infants (*P* were 0.219 and 0.998, respectively) [22]. Another study of 387 infants examining risk factors for PNAC in VLBWI also showed that birth weight and gestational age at birth were not independent risk factors for the development of PNAC (*P* 0.773 and 0.181, respectively) [23], which is consistent with the results of this study. However, lower gestational age at birth and lower birth weight were associated with a higher incidence of early complications in preterm infants, which tend to have more severe symptoms and inevitably lead to a longer hospital stay. Multivariate logistic regression analysis in this study showed that longer hospital stay was an independent risk factor for PNAC (*OR*, 1.030; 95% *CI*, 1.014 to 1.046). Indirectly, this data suggested that lower gestational age at birth and lower birth weight were strongly associated with the development of PNAC.

### The impact of enteral and parenteral nutrition management on PNAC

The maximum dose of amino acids in the infants of the PNAC and non-PNAC groups in this study was 4.0 g/kg/d and 3.5 g/kg/d, respectively, and this study showed that the maximum dose of amino acids was an independent risk factor for PNAC (*OR*, 5.352; 95% *CI*, 2.355–12.16). A study by Sung et al. [24] showed that high doses of amino acids intake in the early postnatal period (3.0 g/kg/d) increased the risk of refeeding syndrome, such as electrolyte disturbances in extremely low birth weight infants. Vileisis et al. [25] performed a prospective controlled study comparing the effects of two different levels of amino acids intake (2.3 g/kg/d vs. 3.6 g/kg/d) on liver function, which showed that increased levels of amino acids intake in PN was associated with the early onset of PNAC and higher bilirubin levels in infants, suggesting that higher level of amino acids intake during PN can directly damage hepatocytes and affect bile excretion from the damaged liver, leading to hepatocellular cholestasis. Data from another retrospective study on factors influencing the progression of PNAC showed a higher incidence of PNAC in infants with an intake of parenteral amino acids > 3.5 g/kg/d compared to those with an intake of < 3.5 g/kg/d (15.1% vs. 6.5%) [22]. This may be attributed to the fact that some of the amino acids in PN, such as methionine, tryptophan and phenylalanine, which considered hepatotoxic, causing cholestasis by directly damaging the tubular membranes of hepatocytes [26]. SMOF is well tolerated in patients consisting of 30% medium chain triglycerides, 30% soybean oil, 25% olive oil and 15% fish oil. ω-6 to ω-3 ratio of long-chain polyunsaturated fatty acids (LCPUFAs) is 2.5:1, which is lower than the 7:1 ratio of medium and long chain fat emulsions. The phytosterol content is lower, while vitamin E and α-tocopherol content is higher in SMOF. SMOF combines the advantages of a variety of oils and is currently considered to be a more balanced fat emulsion. The results of this study showed 13.7% incidence of PNAC in infants receiving SMOF compared to 21.2% in infants receiving medium and long-chain fat emulsions (*P =* 0.002). The results of the multifactorial logistic regression analysis in this study showed that SMOF was a protective factor for PNAC (*OR*, 0.358; 95% *CI*, 0.193 to 0.663). Jiang et al. [27] performed a randomized controlled trial on the effect of SMOF and medium/long chain fat emulsion in 160 neonates after intestinal surgery, which showed that infants receiving SMOF tolerated lipids better and were better protected against PNAC compared to those receiving medium/long-chain fat emulsion, and this protection was more pronounced in infants on intravenous nutrition lasting more than 4 weeks. A retrospective study by Kasirer et al. [28] also found a lower incidence of PNAC in VLBWI with SMOF compared to medium/long-chain fat emulsion (6% vs. 13%; *P* = 0.022). These results are consistent with the present study. It is suggested that SMOF has a better hepatoprotective effect and reduces the incidence of PNAC in infants mainly due to the high content of ω-3 LCPUFAs, which possess anti-inflammatory properties and confer a potent hepatoprotective effect. Olive oil, one of the main components of SOMF, is rich in oleic acid, an ω-9 monounsaturated fatty acid, which not only reduces oxidative stress but also promotes the biosynthesis of ω-3 LCPUFAs in the liver [29]. SMOF contains less phytosterol, which has been shown to inhibit the expression of proteins involved in bile acid and bilirubin excretion in animal studies, leading to cholestasis [30]. SMOF also contains high doses of vitamin E and ɑ-tocopherol, which act as anti-inflammatory and antioxidant agents. It appeared that many components of SMOF are protective factors against hepatotoxicity, which possibly inhibit the development of PNAC in infants.

Studies have shown that breastfeeding promotes growth and development, improves feeding tolerance, reduces oxidative stress and promotes anti-inflammatory responses in preterm infants [31]. It can effectively reduce the incidence of complications, such as BPD, NEC, ROP and PNAC in preterm infants. In this study, multifactorial logistic regression analysis confirmed that breastfeeding (*OR*, 0.297; 95% *CI*, 0.157 to 0.559) was a protective factor for PNAC, and FI (*OR*, 2.581; 95% *CI*, 1.395 to 4.775) was a risk factor for PNAC. The results showed a higher breastfeeding rate in the non-PNAC group compared to the PNAC group (54.4% vs. 27.2%, *P* < 0.001), resulting in a lower incidence of FI, shorter time to achieve total enteral feeding and faster average velocity of weight growth. However, the fewer accumulated days of total calories up to standard 110 kcal/kg/day suggested that energy supply in the early postnatal period for preterm infants in the PNAC group was mainly through PN, particularly via increased amino acid and fat intake, while enteral nutrition was relatively inadequate, leading to a longer duration of PN with an increased risk of PNAC.

Therefore, the management of postnatal enteral and parenteral nutrition of VPI or VLBWI should be balanced by increasing breastfeeding rates, promoting feeding tolerance, avoiding excessive amino acid intake and infusing SMOF, thus, reducing the incidence of PNAC.

### Early comorbidity in preterm infants in relation to PNAC

Lower gestational age at birth and birth weight of preterm infants were associated with poorer neurological development, poorer organ and tissue development and higher incidence of EUGR [32]. In this study, a multifactorial logistic regression analysis revealed that EUGR (*OR*, 2.396; 95% *CI*, 1.255 to 4.572) was an independent risk factor for PNAC, and the PNAC group required longer duration of invasive and non-invasive ventilation, total oxygen support, and had a higher incidence of RDS, hsPDA and LOS (*p* < 0.05 for all). It is suggested that the infants in the PNAC group were severely ill, and the management of enteral nutrition during the early postnatal period is more likely to be neglected, resulting in inadequate enteral nutritional support and a longer time to reach total enteral feeding, yet enteral nutrition was essential to avoid EUGR in preterm infants [33]. Moreover, the duration of PN was prolonged due to inadequate enteral nutrition support, and a longer duration of PN infusion increased the risk of developing PNAC, forming a vicious circle of EUGR and PNAC. Multifactorial logistic regression analysis in this study also found that surgically treated NEC (*OR*, 11.300; 95% *CI*, 2.127 to 60.035) was an independent risk factor for PNAC and contributed most to the progression of PNAC. A retrospective study on factors influencing PNAC in 114 cases of VLBWI (including 27 cases in the PNAC group and 87 cases in the non-PNAC group) found that NEC was an independent risk factor for PNAC [34]. A retrospective study by Kim et al. [35] examining factors influencing cholestasis in VLBWI showed that both stage II or higher NEC and bowel surgery were independent risk factors for PNAC, which is consistent with the results of this study. One study was conducted on NEC concerning postnatal growth at hospital discharge and 2 years later in extremely low birth weight infants by Hong et al. [36] in a total of 9,171 infants, which showed that the incidence of severe growth retardation at discharge was higher in both infants with NEC and those with surgically treated NEC compared to those without NEC (56% vs. 36% and 61% vs. 36%). A study reports a 5-fold increased risk of EUGR once NEC is diagnosed, regardless of surgical treatment [37]. This is related to the fact that prolonged fasting after the occurrence of NEC and clinicians tend to be very cautious about the indication for reopening the breastfeeding after fasting. Surgically treated NEC can lead to short bowel syndrome, disruption of the intestinal mucosal barrier, dysbiosis, intestinal bacterial translocation and absorption of toxins [38, 39], and these factors combined with EUGR collectively increased the risk of PNAC. Therefore, enteral nutrition in preterm infants (VPI or VLBWI) should be prioritized without increasing the incidence of NEC, especially avoiding surgical NEC and reducing the incidence of EUGR [4]. Tailored management of enteral nutrition in VPI play an important role in reducing the incidence of PNAC.

This study collected data from a multicenter observational survey and analyzed the risk factors associated with the development of PNAC in VPI or VLBWI. With a large sample size and more convincing evidence, we provided a further scientific basis for the prevention of PNAC. This study had some limitations. Firstly, the study missed several parameters, including the duration days of fasting of preterm infants in the two groups. However, analyzing the incidence of FI and the time to achieve full enteral feeding could have compensated for the missing parameters. Secondly, the study did not explore the effect of dose of dextrose on PNAC, in the literature, it has been reported that excessive intravenous dextrose intake also increases the risk of developing PNAC [40]. Third, the factors affecting the onset of PNAC may be different in different PN durations, and this study did not in-depth analysis of the effect of different PN durations on the onset of PNAC. The next step will be to conduct a follow-up study to clarify the impact of PNAC on the long-term prognosis of preterm infants.

In summary, the incidence of PNAC can be reduced by increasing breastfeeding for VPI or VLBWI, using SOMF, avoiding excessive amino acid intake, promoting feeding tolerance, preventing severe NEC requiring surgery and reducing the incidence of EUGR.

## Data Availability

All the data used in this study are available from the corresponding author upon reasonable request.

## Abbreviations

VPI: very preterm infants
VLBWI: very low birth weight infants
NICU: neonatal intensive care unit
CHNN: Chinese Neonatal Network
PN: parenteral nutrition
NEC: necrotizing enterocolitis
LOS: late-onset sepsis
BPD: bronchopulmonary dysplasia
PNAC: parenteral nutrition-associated cholestasis
EUGR: extrauterine growth retardation
PMA: postmenstrual age
RDS: respiratory distress syndrome
hsPDA: hemodynamically significant patent ductus arteriosus
EOS: early-onset sepsis
ROP: retinopathy of prematurity
MBDP: metabolic bone disease
FI: feeding intolerance
LCPUFAs: long-chain polyunsaturated fatty acids
MCT: medium-chain triglycerides
LCT: long-chain triglycerides
