# Correction: Analysis of risk factors for parenteral nutrition-associated cholestasis in preterm infants: a multicenter observational study

**DOI:** 10.1186/s12887-023-04116-9

**Published:** 2023-06-17

**Authors:** Ya-sen Wang, Wei Shen, Qing Yang, Rong Lin, Li-xia Tang, Rui-miao Bai, Dong Yang, Juan Zhang, Yi-jia Zhang, Wen-ting Yu, Shi-rong Song, Juan Kong, Si-yu Song, Jian Mao, Xiao-mei Tong, Zhan-kui Li, Fan Wu, Xin-zhu Lin

**Affiliations:** 1 Department of Neonatology, Women and Children’s Hospital, School of Medicine, Xiamen university, Xiamen, 361003 China; 2 Xiamen key laboratory of perinatal-neonatal infection, Xiamen, China; 3 Xiamen Clinical Research Center for Perinatal Medicine, Xiamen, China; 4 Department of Neonatology, Northwest Women and Children’s Hospital, Xian, 710061 China; 5 Department of Pediatrics, Peking University Third Hospital, Beijing, 100191 China; 6 Department of Pediatrics, Shengjing Hospital of China Medical University, Shenyang, 110000 China; 7 Department of Neonatology, The Third Affiliated Hospital of Guangzhou Medical University, Guangzhou, 510150 Guangdong China

**Correction**: ***BMC Pediatr*23, 250 (2023)**

10.1186/s12887-023-04068-0

Following the publication of the original article [1], the authors identified errors in the author list as stated below.

- “^†^Ya-sen Wang, Wei Shen, Rui-miao Bai, Juan Zhang, Wen-ting Yu, Juan Kong contributed equally to this work”, however, in the published version, this was incorrectly captured as “^†^Ya-sen Wang, Wei Shen, Juan Zhang, Yi-jia Zhang, Shi-rong Song, Juan Kong contributed equally to this work.”
- Jian Mao, Xiao-mei Tong, Zhan-kui Li, Fan Wu, Xin-zhu Lin are co-corresponding author on this paper, but in the published version, only Xin-zhu Lin has been captured as such.
- The affiliations 2 and 3 were incorrectly captured as: “^2^Xiamen key laboratory of perinatal-neonatal infection, (none)Helping to remove the bracketed content, please, Xiamen, China”; “^3^Xiamen Clinical Research Center for Perinatal Medicine, (none)Helping to remove the bracketed content, please, Xiamen, China”. These should have been captured as “^2^Xiamen key laboratory of perinatal-neonatal infection, Xiamen, China”; “^3^Xiamen Clinical Research Center for Perinatal Medicine, Xiamen, China”.

The original article has been corrected.
